# Pulpless Teeth—Should They Be Extracted?

**Published:** 1920-11

**Authors:** Thomas Cowling


### PULPLESS TEETH—SHOULD THEY BE EXTRACTED?

BY THOMAS COWLING, D.D.S.

In these days of radicalism dentistry can not boast of freedom from those elements which glory in drastic and revolutionary methods of practice. In some quarters moderation is considered old-fashioned and as such must needs be discarded. Everything new, destruction for old theories and practices, however satisfactory they may have been—this, we sometimes are forced to believe, is the basis of our new teaching. As an illustration of this tendency we have those who advocate and practice the extraction of every tooth in which pulp treatment is involved.

Many dentists are now coming to the conclusion that such practice is too radical and that the results obtained do not warrant its continuance. Others still cling to the belief that the early removal of all teeth in which pulp treatment of any sort whatsoever is required, is necessary if the patient's health is to be properly safeguarded. That there should be such divergence of opinion on such an important question shows that our investigations have been incomplete and hurried, otherwise a finality of opinion would have been established one way or another. The present status of the subject is not flattering to our profession. Our patients suffer the needless loss of teeth, perhaps, and we are undecided as to the right thing to do. Unquestionably this is the biggest problem facing the medical and dental profession to-day.

The *International Journal of Orthodontia* in its June issue takes up, in a very able manner, the defense of the pulpless tooth. It is pointed out that the advocates of extraction have, as a rule, in their enthusiasm to establish a case, set aside evidence that could be produced in favor of retaining pulpless teeth, and the only evidence considered was the "case reports" of a certain number of patients in whom it was supposed that systemic infection could be traced to pulpless teeth with infection at the ends of the roots. While it is true that some patients with systemic infections have improved after the removal of pulpless teeth, it is also equally true that a large percentage of systemic infections did not improve. Many cases of rheumatic tendency, arthritis, etc., have improved after the pulpless teeth are extracted, but this does not prove that the infection from the tooth was the only infection in the body related to these disorders. There may be many other sources of infection. Very often when this improvement occurs, it is the result of the "change of balance" between the number of micro-organisms in the system of the individual and the number of micro-organisms which the leucocytes in the system can take care of. Improvement at the time, or shortly after, only proves one of two things. First, by the removal of the teeth some of the sources of infection in the body have been eliminated, which changes the "balance" for a certain period of time. Second, by the removal of the teeth some of the blood vessels in the immediate area of infection have been opened and some of the germs which were present around the tooth have gained entrance into the blood stream and have produced a change therein similar to bacterial vaccine, and consequently increased the action of the leucocytes for a period. After the removal of a pulpless tooth it is very often observed that the individual has a rise of temperature, increased pulse, which is generally caused by germs entering the blood stream and which act upon the blood contents in a beneficial manner. After the recovery of the first shock from the extraction and the rise in temperature, the individual immediately improves. Such a condition proves only that the tooth infection may have been only one of the causes, because it is generally observed that such an improved condition is not permanent. Herein lies the disappointment which follows extensive extractions.

Most of us are apt to look for greatly improved conditions in rheumatic cases where the pulpless teeth have been suspected as the inciting cause, and have been extracted. In the editorial of the *International Journal* it is pointed out that there may be infection anywhere in the alimentary tract, in the liver, the gall bladder, kidneys, fallopian tubes, prostate glands, or any other part of the anatomy. It is also to be remembered that a great many more rheumatic conditions associated with systemic disturbances are the result of gonorrhea, than are caused by pulpless teeth. It is just possible that the infection at the root of a tooth may be in some cases secondary, the initial infection being in the tonsils or other organs. This has yet to be investigated.

Even if germs are found in the granuloma at the root of a tooth, it is, in the editor's opinion, no positive evidence that the granuloma is the cause of the systemic infection in the individual. Tubercule bacilli may be found in the lungs of many individuals who are not suffering from tuberculosis. The advocate of extraction says that if the germ is found in the granuloma the tooth should be removed as a prophylactic measure. In view of the fact that every organ of the body is subject to infection at all times, subject to the invasion of micro-organisms which are eliminated in course of time, his contention if applied generally would mean that any organ of the body, if infected, must be removed. The results would prove rather disastrous.

One of the most serious aspects of this extraction epidemic is that many operators are not satisfied with the complete extraction of the offending teeth, but insist upon their removal surgically, which means that a considerable portion of the alveolar process comes away too; this is for the purpose of getting rid of all possible infection. For such cases it becomes an exceedingly difficult task to make an artificial restoration.

The opposition to the present radical policy regarding pulpless teeth is not confined to this country alone. Away from Australia comes a plea for the early reconsideration of the whole problem. In the June issue of the *Commonwealth Dental Review,* the editor expresses regret that hysteria should have taken the place of common sense. He says: "How a new doctrine of this kind (the extraction of all pulpless teeth) can suddenly impress itself so forcibly upon dentists far and wide that they find themselves preaching it to their patients and putting it into practice on every occasion, is somewhat difficult to understand. It is possible that dentists are being too largely influenced by medical men in this matter."

If we could but arouse the interest of some of our best thinkers, who, by the way, are seldom heard from either from the platform or press, we could very speedily destroy the present policy of "extraction without discussion."—*Oral Health.*

